# Effects of LED Light Illumination on the Growth, Digestive Enzymes, and Photoacclimation of *Goniopora columna* in Captivity

**DOI:** 10.3390/ani12030306

**Published:** 2022-01-26

**Authors:** Chiu-Min Cheng, Yu-Rong Cheng, Hsuan-Yu Lin, Wei-Ting Sun, Chih-Hung Pan, De-Sing Ding

**Affiliations:** 1 Department and Graduate Institute of Aquaculture, National Kaohsiung University of Science and Technology, No. 142, Haijhuan Rd., Nanzih District, Kaohsiung 811, Taiwan; cmcheng@nkust.edu.tw (C.-M.C.); a19992716@gmail.com (H.-Y.L.); 1051537103@nkust.edu.tw (W.-T.S.); chpan@nkust.edu.tw (C.-H.P.); 2 Department of Fisheries Production and Management, National Kaohsiung University of Science and Technology, No. 142, Haijhuan Rd., Nanzih District, Kaohsiung 811, Taiwan; yrcheng@nkust.edu.tw

**Keywords:** illumination, digestive enzymes, *Goniopora columna*, growth, photoacclimation

## Abstract

**Simple Summary:**

Coral aquaculture is a new industry, which is of great importance to the sustainable development of coral reefs and meeting commercial needs. Light sources are crucial for the growth of corals because zooxanthellae provide them with essential nutrients through photosynthesis. Different corals and zooxanthellae have different photoacclimation characteristics; therefore, selecting a suitable light wavelength remains the critical inhibitor of coral maintenance in marine aquariums. Accordingly, this study investigated the effects of different light wavelengths and feeding of *G. columna*. The results showed that blue light (440–470 nm) and purple light (400–430 nm) increased the protease and body protein in corals, and the growth and survival rate also increased. In summary, *G. columna*’s efficient cultivation can use 400–470 nm wavelengths as the primary source of illumination.

**Abstract:**

*Goniopora columna* is a stony coral valued for its reef-building potential and its unique appearance. Thus, identifying the optimal culture conditions for *G. columna* would enable efficient cultivation and prevent the illegal exploitation of marine resources. Light sources are crucial for the growth of corals because zooxanthellae provide them with basic nutrients through photosynthesis. Different corals and zooxanthellae have different photoacclimation characteristics; therefore, selecting a suitable light wavelength remains the key inhibitor of coral maintenance in marine aquariums. Accordingly, this study investigated the effects of different light wavelengths on *G. columna*. It was illuminated for 6 or 12 h a day under white light, yellow light, red light (LR), green light (LG), blue light (LB), or purple light (LP) for 8 weeks. During the experiment, R(R; i.e., a formula feed that combines sodium alginate, protein and probiotics) of 5% (*w/v*) of *G. columna* tissue and skeletal dry weight was fed every day. Coral polyps were counted, zooxanthellae density, chlorophyll a concentration, specific growth rates, and survival rates were calculated; polyp stretching and contractile behaviors were observed; and body composition and digestive enzyme activity were analyzed. LB or LP (but not LG or LR) illumination for at least 6 h per day significantly promoted the growth, survival, protein content, and protease activity of the *G. columna* specimens. Furthermore, coral polyp extension reached 100% after 30 min of LP and LB light irradiation. Although no significant differences in the zooxanthellae density or chlorophyll a concentration were noted under various light wavelengths, significant reductions were detected in the absence of light. To achieve energy-efficient coral aquaculture with regard to *G. columna* cultivation, 6 h of LB or LP illumination per day can improve the growth.

## 1. Introduction

Corals are not only pivotal in marine ecology but are also popular in aquariums. Marine aquarium owners number 1.5–2 million worldwide, and the corals they own are mainly obtained from coral reefs. According to records from the Convention on International Trade in Endangered Species of Wild Fauna and Flora (CITES), the trade volume of *Goniopora columna* was 7.2 million (18% of the total coral trade) between 2010 and 2020 (CITES, 2021). Therefore, the efficient captive breeding of *G. columna* can increase demand for aquariums and reduce the destruction of coral reefs. *Goniopora columna* is a scleractinian coral native to the Indian Ocean and Pacific Ocean [1]. The polyps of *G. columna* are large, long (>5 mm) and easy to observe. They are commonly seen in intertidal or muddy waters coral reefs, commonly known as flowerpot coral. Tubular creatures (polyps) with conical projections in the center of their tentacles have both ecological and ornamental roles. Therefore, artificially cultivated *G. columna* presents considerable potential for commercial applications.

Ding et al. [2] found that *G. columna* can only feed when there is light, which can promote its growth. However, there is no research on the light wavelength and light time suitable for coral growth, which will be of great help to coral large-scale aquaculture [2]. Light is a key factor in coral productivity, physiology, and ecology [3,4,5]; under suitable lighting conditions, corals can grow more quickly and efficiently. The zooxanthellae in the coral endoderm can generate basic energy for coral physiology or growth through photosynthesis, and appropriate lighting is critical for the growth and survive of coral in captivity [6]. The energy-rich photosynthates produced by symbiotic zooxanthellae feed corals and coral metabolic waste offers nutrients essential for symbiont tissue growth [7]. Symbionts do not generate a coral tissue, but live inside coral cells. Symbiont growth allows cell duplication and the maintenance of these populations in the same coral tissue or populates new growing coral tissue. The light spectrum plays a critical role in the biology of symbiotic corals. It affects zooxanthellae density, chlorophyll a content, and coral survival [8], enhances calcification [9] and induces the contraction and extension of polyps (Levy et al., 2003) [10]. The expansion and contraction of coral polyps are critical behaviors, which are related to light [11,12], capture of prey [13,14], removal of sediment [15,16], increase in oxygen, and diffusion of waste [17,18]. This pattern of polyp expansion and contraction differs depending on the coral species; for instance, species containing high-density zooxanthellae may expand under light conditions conducive to photosynthesis, whereas those containing little to no zooxanthellae may contract under the same conditions [12]. Enriquez et al., found that coral skeleton is fundamental to amplify the light environment of corals in hospite thanks to multiple scattering of light on coral skeleton [19,20]. Thus, low pigmented corals with retracted polyps can provide high local levels of irradiance within the tissue to the symbionts. In addition, when there is light, zooxanthellae photosynthesize to produce oxygen provided to corals to help digest food. Moreover, some predatory coral polyps containing high-density zooxanthellae have dual functions of capturing prey and performing photosynthesis. According to the research of Falkowski et al. [21], the carbon produced by zooxanthellae can differ by up to 60% in shady or sunny reef areas. *Stylophora pistillata* can provide basic carbon for growth through zooxanthellae, and corals growing in shaded reef areas must rely on feeding for basic nutrients [21]. In addition, a previous study found that Acropora intermedia can cause bleaching or death under strong sunlight exposure, and if there is an environment with high sediment, mortality can be reduced. Therefore, not all light sources are suitable for coral growth [22].

*Goniopora* sp. is a carnivore; in addition to obtaining nutrients from zooxanthellae, it must catch prey in its environment. The gastrovascular cavity resides in the main organ of corals’ digestive systems. Food entering the digestive system must be decomposed through mechanical or chemical processes before it can be digested and absorbed. The chemical breakdown is aided by factors such as digestive enzymes. Therefore, digestive enzyme activity can determine the ability of a species to use different nutrients and effectively fulfill dietary needs [23]. Raz-Bahat et al. detected chymotrypsinogen in *Stylophora pistillata*’s digestive enzyme, actinopharynx, peristome, and mesenterial filaments, which is a key digestive organ containing various gland cells and digestive enzymes [24]. Ding et al. [2] found that digestive enzymes in *G. columna* changed due to feeding or its biological clock. Several mesenterial filaments were observed on a polyp, but whether polyp stretching is related to digestion remains unclear. In addition, environmental factors, such as temperature, light intensity, and food, may alter the body composition [25,26,27]. However, whether light wavelengths affect the body composition of coral also remains undetermined. Therefore, this study investigated the optimal illumination condition for *G. columna* and determined whether illumination affects its growth, survival, zooxanthellae density, chlorophyll a concentration, body composition, and digestive enzyme activity.

## 2. Materials and Methods

### 2.1. Biological Material

The *G. columna* (one hundred colonies) coral samples used in the experiment were taken from the TCK coral farm of Kaohsiung, Taiwan *G. columna* is a cultured coral, cites number No. FTS507W0153796. The corals were kept in a glass tank (60 × 35 × 30 cm) with a recirculating filtered seawater system. Moreover, a water pump was installed to remove the mucus on the coral’s surface, thereby preventing tissue necrosis [28]. After 2 months of acclimation and self-repair, healthy corals were segmented into a colony containing 5 polyps and then stuck on a rough cornerstone with coral glue. Each colony weighed 1.72 ± 0.54 g. Each group contained 10 colonies. All experiments were repeated three times, and thus each group totally involved 30 colonies (*n* = 30). After three days of coral tissue repair, the polyps were examined at full extension, and the experiment commenced. The feed used in this study is based on Ding et al. [2]. The feed contained a mixture of intact and hydrolyzed marine and terrestrial ingredients (commercial-in-confidence formulation, details not provided). Artificial polyunsaturated fatty acids (PUFAs) are rich in animal protein (R; i.e., a formula feed that combines sodium alginate, protein and probiotics). During the experiment, corals were fed 5% (*w/v*) of their skeletal dry weight once a day.

### 2.2. Experimental Conditions

We used HME Block 2 Series light-emitting diode (LED) light sources. Lamps were installed 30 cm above the water surface of each glass tank (60 × 45 × 30 cm^3^). Six types of LEDs irradiating white (LW), yellow (LY), red (LR), green (LG), blue (LB), and purple (LP) light were used, and a non-illumination group (C) served as the control. The lighting durations were set as either (i) 6 h (8:00–14:00; short illumination group) or (ii) 12 h (8:00–20:00; long illumination group; [29]). A total of 13 groups were involved in this study, and each experiment was repeated three times. PAR was measured using Apogee Instruments MQ-510 underwater quantum meter (USA). Details are presented in Table 1. All groups’ photosynthetically active radiation (PAR) and water quality were measured daily during the experiment. During the experiment, the water quality was controlled at temperature 26.41 ± 0.04 °C, salinity 35.19 ± 0.42 PSU, pH 8.13 ± 0.36, ammonia nitrogen 0.04 ± 0.01 mg/L, nitrous acid 0.02 ± 0.02 mg/L, nitric acid 0.18 ± 0.02 PPM, calcium 412.69 ± 16.36 PPM, magnesium 1304.54 ± 19.16 PPM and phosphate 0.02 ± 0.01 PPM. The water quality data during the experiment are shown in Table 2. The experiment was conducted over 8 weeks, at the conclusion of which the coral weight, polyp count, zooxanthellae density, chlorophyll a concentration, body composition, and digestive enzyme content of the coral were measured.

### 2.3. Determination of Polyp Count, Growth, and Survival Rate

*G. columna* growth was determined based on the polyp count and the total specific growth rate (SGR) was calculated as described by Tilstra et al. [30], Rocha et al. [31] and Schutter et al. [32] Regarding the polyp counting operation, the *G. columna* colonies had a large polyp that could be observed directly with the macro observation; it was recorded with a Canon EOS 750D camera. The photographs were captured and the calculations were made weekly over 8 weeks. We also determined the dry weights of the coral tissue and skeleton. Specifically, for each porous foundation stone, algae were brushed away, and the surface of the coral was dusted off before weighing. Coral weight was measured using an electronic balance that had been reset to zero. The coral was weighed weekly over the study period, and its SGR was computed using the following formula:

$$\mathrm{SGR}\;(\%\;{\mathrm{Day}}^{-1})=\left(\frac{In\left(wf\right)-In\left(wi\right)}{\Delta t}\right)\;\times \;100$$

where *wf* is the final weight of the *G. columna*, *wi* is the initial weight of the *G. columna*, respectively, expressed in grams (g), and Δ*t* is the experimental time (days), SGR was simplified to day^−^^1^.

At the end of the 8-week experiment, each coral was assessed to be either dead or alive. The definition of “alive” was that the coral structure still contained a polyp [33]. To calculate the coral survival rate, we used the following formula:

Survival rate (%) = (final number of living specimens ÷ number of initial specimens) × 100

### 2.4. Photoacclimation of G. columna

Before the experiment, *G.* *columna* specimens were taken from the main tank, placed in separate fish tanks (20 × 20 × 15 cm^3^), and allowed to reside in the dark. Each of the 7 groups comprised 20 colonies, and each experiment was repeated 3 times; a total of 420 colonies were subjected to the experiment. After 2 h of acclimatization to complete darkness, the specimens were either left in the dark or illuminated with LW, LY, LR, LG, LB, or LP light. There was no feeding during the experiment. The polyps’ stretching and contractile behaviors were recorded every minute for 30 min on the Canon EOS 750 D camera and rated on the 5-point scale (0–4 points) proposed by Lasker and Levy [10] (Table 3). All polyps were completely retracted in the dark. Thus, their scores were 0. A score of 1, 2, 3, and 4 points represented 25%, 50%, 75%, and 100% extension, respectively. These observations were conducted to determine which light source was most suitable for *G.* *columna*. Final analyses were performed only on the corals exhibiting 0% and 100% extension.

### 2.5. Analysis of Coral Body Composition

After the experiment, the *G. columna* tissues were homogenized and sonicated, and the protein concentration was tested using the Bradford protein assay kit (Amresco, Solon, OH, USA), with bovine serum albumin serving as the protein standard. For fat content analysis according to standard methods [34], lipids were extracted from *G. columna* by using hexane; subsamples were then transferred to test tubes and evaporated to dryness. The total lipid weight was determined (±0.0001 g), and the derived weight values were converted to micrograms (1 g = 1 × 10^−6^ µg). We calculated each lipid with the following formula:

(1) $$\mathrm{Lipid}=\frac{Wi-Wo}{S}\times 100$$

where *Wo* represents the constant weight of the aluminum cup (g), *Wi* represents the weight of the extracted oil contained in the aluminum cup (g), and *S* represents the sample weight (g). Carbohydrates were measured using the method proposed by Bishop [35] and Tietz [36], with glucose serving as reference material. Absorption values of 505–660 nm were used to determine glucose content. The formula for glucose content derivation is expressed as follows:

$$\mathrm{glucose}\left(µ\frac{g}{\mathrm{mg}}\right)=\frac{A\left(\mathrm{Samplewithcolorimetrictest}-\mathrm{sample}\right)}{A\left(\mathrm{Standardtube}\right)}\times \mathrm{Glucosestandardconcentration}\left(µ\frac{g}{\mathrm{mg}}\right)$$

### 2.6. Analysis of Digestive Enzymes

After the experiment, digestive enzyme analysis was performed. Digestive enzyme detection method is referred to Sun et al. [37]. Lipase and protease extraction was performed with 10 mM sodium citrate buffer (pH 7.0) in a low temperature environment. Each coral was first rinsed in the buffer solution, after which the buffer solution was added at 10 times the volume of the coral specimen. The *G.* *columna* was placed on ice for homogeneous grinding and then centrifuged (4 °C, 10,000× *g*, 10 min). The supernatant was collected and stored at −20 °C. Refer to the method of [37] for protease detection. Protease detection, 1 mL of casein was added to 0.5 mL of enzyme extract, and the mixture was incubated for 15 min, after which 1.5 mL of 10% trichloroacetic acid was added. Subsequently, the mixture was centrifuged at 6000× *g* at 4 °C for 10 min, the supernatant was collected, and 5 mL of 0.55 M Na_2_CO_3_ and 1 mL of Folin’s phenol staining reagent were added; the absorbance value at 680 nm was measured. Lipase content analysis was conducted using the method proposed by Borlongan [38]. Lipase detection, 1.5 mL of olive oil was added to 1.5 mL of Tris–HCl (0.1 M buffer, pH 8.0) and 1 mL of enzyme extract, and the mixture was then placed at 37 °C for 6 h with shaking. To terminate the reaction, 95% alcohol was added. Thymolphthalein containing 0.9% alcohol was used as the indicator and the mixture was then titrated with 0.01 N NaOH until the solution color turned brown. Amylase content analysis was executed using the method presented by Bernfeld [39]. Specifically, 0.05 M phosphate buffer solution (pH 7.0) was added to 1 mL of 2% (*w/v*) starch solution, and the mixture was maintained at 25 °C for 5 min. Subsequently, an enzyme extract was added, and the mixture was reacted at 20 °C–60 °C; moreover, 2 mL of dinitrosalicylic acid reagent was added. The reaction was stopped in a boiling water bath for 5 min, and the mixture was cooled. The absorbance was then measured at 520 nm (maltose as standard). Amylase activity was evaluated as maltose content per milligram of protein per minute. At the end of the experiment, the means and standard deviations (SDs) were calculated.

### 2.7. Analysis of Zooxanthellae Density and Chlorophyll a

At the end of the experiment, the *G.* *columna* tissues were homogenized ground, and the number of zooxanthellae in *G.* *columna* were observed and calculated with a blood cell counter according to Titlyanov et al. [40]. Zooxanthellae density was expressed as number per polyp. Chlorophyll a concentration was determined according to the methods of [10,40]. In brief, fresh coral tissue (0.5 g) was homogenized, and then 10 mL of 90% acetone was added to extract the chlorophyll a. Finally, the tissue was left to stand for 24 h at 4 °C in pitch-black conditions. Absorption spectra were measured at 630 nm and 664 nm using a Hitachi U-2000 spectrophotometer (Tokyo, Japan). Using the equations developed by Jeffrey & Humphrey [41], the chlorophyll a concentration was calculated immediately as micrograms per gram of colony tissue wet weight.

### 2.8. Statistical Analysis

Data were obtained from 3 independent experiments, and the final results are presented as means ± SDs. Two-way analysis of variance, Homogeneity of variance test and Duncan’s multiple range test were conducted to determine statistically significant effects (*p* < 0.05) on coral growth, survival, digestive enzymes, body composition, and zooxanthellae density and chlorophyll a concentration. All analyses were performed using IBM SPSS Statistics for Windows, version 20 (IBM Corp., Armonk, NY, USA).

## 3. Results

### 3.1. Effects of Different Types of Illumination on G. columna Growth and Survival

**Figure 1:**
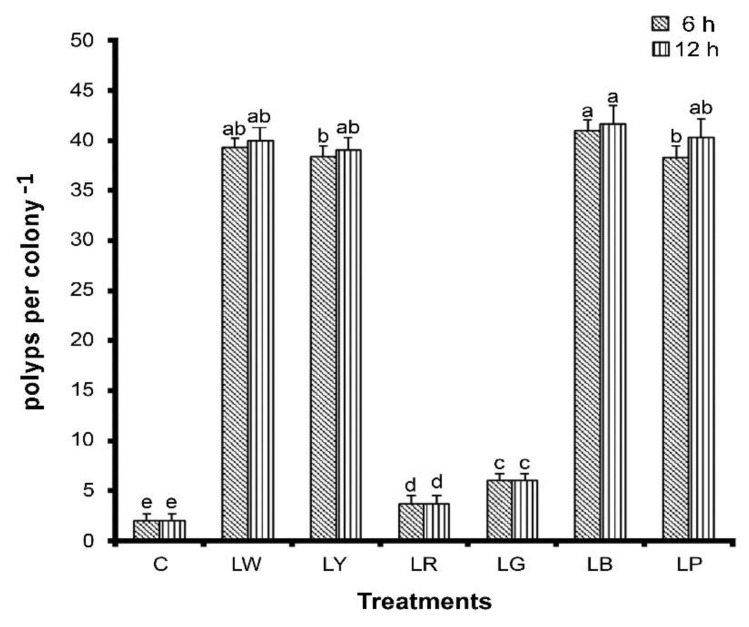
Number of *G. columna* polyps after illumination under different light sources. *Goniopora columna* specimens were treated with LW (444–696 nm), LG (500–540 nm), LY (570–590 nm), LB (440–470 nm), LR (620–650 nm), or LP (400–430 nm) for 6 or 12 h per day. C was in the dark. After 8 weeks, polyps were counted. Bars represent ± SD (*n* = 30). Letters represent significant differences among groups (*p* < 0.05).

**Figure 2:**
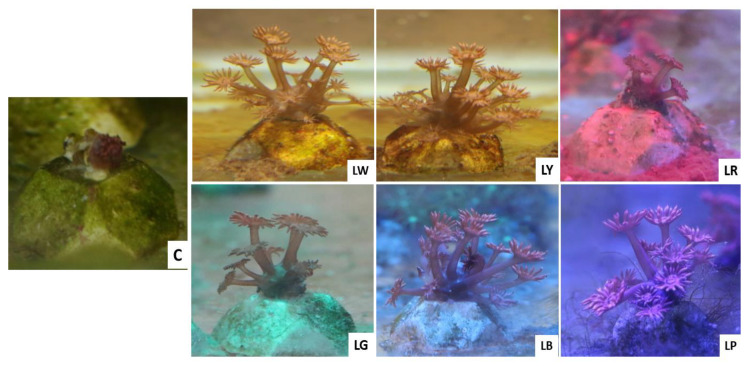
Photoacclimation of *G. columna* illuminated by various light sources. *Goniopora columna* specimens were exposed to LW (444–696 nm), LG (500–540 nm), LY (570–590 nm), LB (440–470 nm), LR (620–650 nm), or LP light (400–430 nm) for 30 min. The stretching and contractile behaviors of the polyps were captured using a Canon EOS 750D camera.

**Figure 3:**
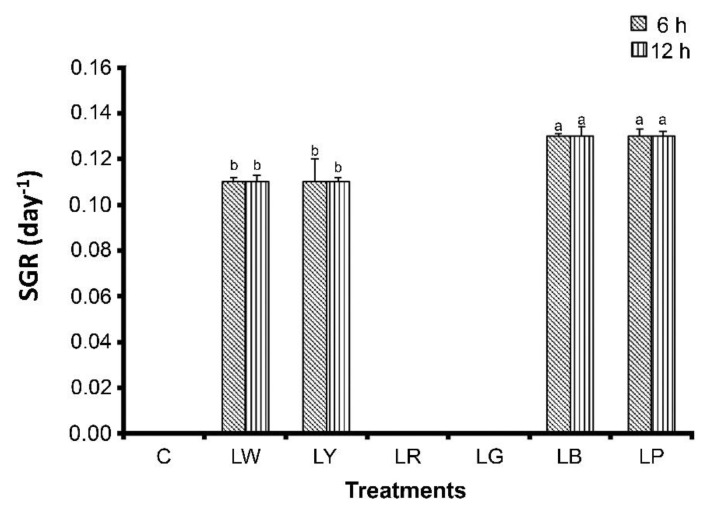
Growth of *G. columna* after illumination under different light sources. *Goniopora columna* specimens were treated with LW, LG, LY, LB, LR, or LP for 6 h or 12 h per day. The control group (C) was maintained in dark conditions. The specimens were weighed once a week for 8 weeks, and the specific growth rates (SGRs) were calculated. Different letters indicate significant differences among groups (*p* < 0.05). Values are expressed as means ± standard deviations (*n* = 30).

**Figure 4:**
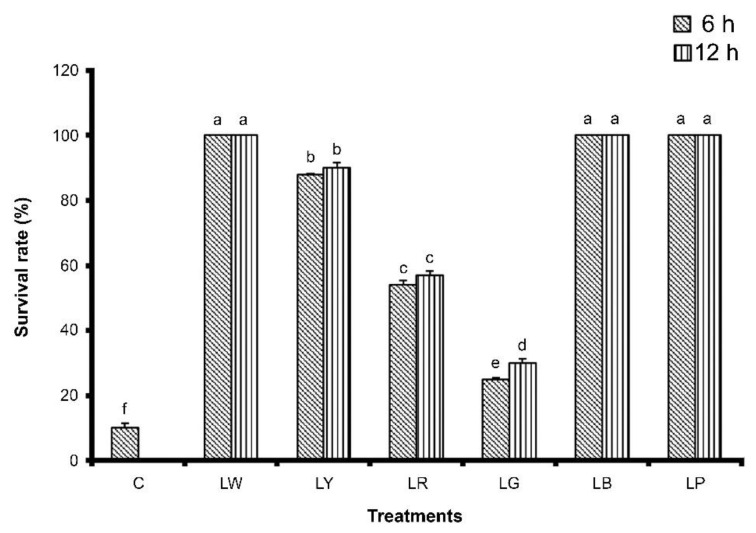
Survival of *Goniopora columna* after illumination under different light sources. *Goniopora columna* specimens were exposed to LW (444–696 nm), LG (500–540 nm), LY (570–590 nm), LB (440–470 nm), LR (620–650 nm), or LP (400–430 nm) light for 6 or 12 h per day over 8 weeks. The survival rates were calculated. Different letters indicate significant differences among groups (*p* < 0.05). Values are expressed as means ± standard deviations (*n* = 30).

To assess whether illumination affects coral growth and survival, we cultured *G. columna* specimens under six illumination conditions (LW, LY, LR, LG, LB, and LP); in addition, some of the specimens were not cultured under illumination, and they constituted the control group. After 8 weeks of cultivation, *G. columna* growth was evaluated by examining polyp count and SGRs. As illustrated in Figure 1, the numbers of polyps in the LB group were 41.00 ± 1.03 and 41.67 ± 1.78 under 6 h and 12 h of illumination, respectively, significantly more than those in the other groups (*p* < 0.05). The control group had the lowest number of polyps, with only two polyps remaining after 8 weeks. We observed no significant differences between 6 h and 12 h of illumination. Furthermore, *G. columna* exhibited stunted growth and shrinkage in the LR and LG groups (Figure 2). As displayed in Figure 3, the SGRs of *G. columna* in both the LB and LP groups were 1.18-fold higher than those in the LW and LY groups under both 6 and 12 h of illumination; the LR and LG groups exhibited zero growth. These results demonstrate that light is essential for *G. columna* growth, and LB and LP illumination can enhance the growth of *G. columna*. Figure 4 presents the survival rates of *G. columna* after 8 weeks of cultivation under different illumination conditions. The survival rate in the LW, LB, and LP groups was 100% under 6 or 12 h of illumination per day, and this rate was 1.13-, 1.85-, 4-, and 10-fold higher than those in the LY, LR, LG, and control groups, respectively. No significant differences were observed in the survival rate of *G. columna* cultured under 6 and 12 h of illumination per day, except in the LG group.

### 3.2. Effects of Different Types of Illumination on Coral Body Composition

After 8 weeks of cultivation, the body composition of *G. columna* was determined. The protein content levels in the LP group were 426.61 ± 6.42 and 421.11 ± 2.32 µg under 6 and 12 h of illumination, respectively (Table 4), which were significantly higher than those in the other groups (*p* < 0.05). We observed no significant differences in fat or glucose content among the groups. These results indicate that LP can enhance the protein content but not lipid or glucose content in *G. columna*.

### 3.3. Effects of Different Types of Illumination on Coral Digestive Enzymes

To verify whether illumination affects the digestive enzymes activity of *G. columna* symbionts, we measured the activity of the digestive enzymes of *G. columna* symbionts after illumination for 8 weeks. Table 5 shows that LP group has the highest protease activity, which were 231.37 ± 9.00 U/mg protein and 231.21 ± 5.40 U/mg protein (*n* = 30 colonies) under 6 and 12 h of illumination, respectively, and the control group (in dark) has the lowest protease activity compared with other groups, which was 58.55 ± 5.83 U/mg protein. LP group also has the highest lipase activity, which were 12.05 ± 1.74 U/mg protein and 14.24 ± 1.31 U/mg protein (*n* = 30 colonies) under 6 and 12 h of illumination, respectively, and LG group has the lowest lipase activity, which were 6.04 ± 1.36 U/mg protein and 5.03 ± 1.42 U/mg protein (*n* = 30 colonies) under 6 and 12 h of illumination, respectively. We observed no significant difference in amylase activity between the groups. These results indicate that illumination or non-illumination might affect protease activity of *G. columna* symbionts, and LP can enhance protease and lipase activity but not amylase activity in *G. columna* symbionts.

### 3.4. Effects of Light Wavelength on Coral Photoacclimation

**Figure 5:**
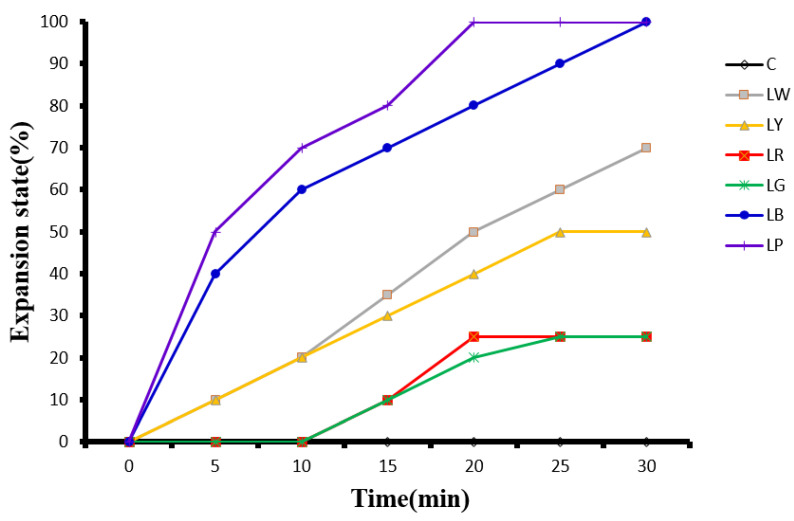
Extension state of *Goniopora columna* polyps after illumination under different light sources. After 2 h of acclimatization to darkness, the specimens were either left in darkness or illuminated with LW, LY, LR, LG, LB, or LP light for 30 min. The stretching and contractile behaviors of the polyps were captured using a Canon EOS 750D camera.

As mentioned, in the experiment, *G. columna* was either left in darkness or illuminated with LW, LY, LR, LG, LB, or LP light for 30 min, and subsequently the stretching and contractile behaviors of the polyps were examined and rated. The polyp patterns observed are presented in Figure 2. Under LW exposure, the polyps extended to 10% (0.18 ± 0.02 cm) after 5 min, 50% (0.84 ± 0.06 cm) after 20 min, and 70% (1.37 ± 0.05 cm) after 30 min. Under both LR and LG exposure, the polyps began to stretch after 10 min and extended to only 25% (0.47 ± 0.01 cm) after 30 min. Under LY exposure, the polyps responded immediately and extended to 50% (1.13 ± 0.06 cm) within 25 min but only to 50% (1.43 ± 0.12 cm) after 30 min. Under LB exposure, the polyps extended to 40% (0.81 ± 0.05 cm) after 5 min and to 100% (2.20 ± 0.21cm) after 30 min. Under LP exposure, the polyps extended to 50% (1.21 ± 0.17 cm) after 5 min and to 100% (2.21 ± 0.09 cm) after 20 min. The polyps in the non-illumination group remained contracted. The maximum rate of extension was 100% for the polyps in the LP and LB groups, 70% for those in the LW group, 50% for those in the LY group, and 25% for those in the LR and LG groups. The control group exhibited 0% extension (Figure 5).

### 3.5. Effects of Light Wavelength on PAR, Zooxanthellae Density, and Chlorophyll a Concentration

To understand the energy efficiency of various light wavelengths used in coral cultivation, PAR was measured daily. Under 6 and 12 h of LB illumination, the PAR was 66.67 ± 0.38 and 68.03 ± 0.42 μmol m^−2^s^−1^, respectively. Under 6 and 12 h of LP illumination, the PAR was 71.03 ± 0.21 and 70.49 ± 0.44 μmol m^−2^s^−1^, respectively, significantly higher than that under exposure to LR over the same durations (44.05 ± 1.01 and 43.98 ± 1.31 μmol m^−2^s^−1^, respectively) and LG (46.35 ± 0.75 and 48.49 ± 1.54 μmol m^−2^s^−1^, respectively). Although the PAR under LW exposure was 102.23 ± 1.01 and 100.58 ± 0.73 μmol m^−2^s^−1^, respectively, higher than that under LB and LP exposure, LW exposure was observed to cause polyp contraction. Therefore, LB and LP illumination were determined to be more suitable for *G. columna* (Table 1). As shown in Table 6, no significant differences in zooxanthellae density (approximately 4.0 × 10^7^ cells m^−2^) or chlorophyll a concentration (approximately 51 µg cm^−2^) were noted under different light wavelength conditions. However, in the control group, the zooxanthellae density decreased to 0.3 × 10^6^ cells m^−2^, and the chlorophyll a concentration dropped to 14 ± 1.33 µg cm^−2^. Therefore, although the content of zooxanthellae and chlorophyll a in *G. columna* were not affected by different light wavelengths, they were notably reduced by the absence of light.

## 4. Discussion

This study demonstrated that light wavelength plays a prominent role in *G. columna* survival and growth. LB and LP were determined to be the best for the extension of *G.* *columna* polyps. They could also enhance the growth and survival of *G. columna*, although at least 6 h of illumination per day was essential for its survival. LB or LP illumination for 6 h per day could enhance *G. columna* protease activity as well as the protein content but did not affect the chlorophyll a concentration or zooxanthellae density. Overall, the results showed that 6 h of LB or LP exposure per day for *G. columna* increased growth. Previous studies found that *G. columna* had better growth when feeding from 06:00 to 12:00, which may also be related to the wavelength of light, and further discussion is needed in the future [2].

The light spectrum is a critical factor in the growth of symbiotic corals. *Maragos* identified a positive relationship between light energy and the growth of *Pocillopora damicornis* and *Pocillopora meandrina* [42]. *Wijgerde* et al. demonstrated that compared with red light, LB leads to higher zooxanthellae density, photosynthesis rates, and coral growth for *Stylophora pistillata* [8]. The present study similarly observed that LB illumination enhanced *G. columna* growth, and the SGR was also significantly higher under LB or LP illumination (400–450 nm) than under exposure to the other wavelengths. Moreover, *G. columna* exhibited stunted growth under daily LR and LG exposure (whether for 6 or 12 h). However, differing from previous research, the different wavelengths did not affect the zooxanthellae density or chlorophyll a concentration in *G.* *columna.* This difference may be attributable to the susceptibility of different types of zooxanthellae to irradiation [43].

*Bessell-Browne* et al. reported that low light conditions can cause bleaching in staghorn coral tissues and the discoloration and death of crustacean coralline algae [44]. Guest et al. [45] transplanted ramets of *G. columna* to different depths (2.2, 7.6, and 8.9 m) in Singapore. One year after the transplantation, fecundity at the depth of 2.2 m had not changed significantly, but bleaching had occurred at depths of 7–9 m. The PAR detected at the 9-m depth was less than 0.6%, whereas that detected at the 3-m depth was greater than 20%. This suggests that the combination of greater depths and low PAR causes bleaching and high *G. columna* mortality. This emphasizes the importance of photosynthetic energy conversion for *G.* *columna*. In the present study, we observed a 10% survival rate in the control group; only two polyps remained. This may be due to the fact that in darkness, the zooxanthellae could not perform photosynthesis to provide basic nutrients to sustain the corals. Therefore, our findings demonstrate that light is critical to the survival of *G.* *columna*. Exposure to short-wavelength light (400–470 nm) for at least 6 h per day exerts the promoted impacts on *G. columna* growth.

The extension of coral polyps is stimulated by factors including light, water flow, and prey [46]. Levy et al. [10] reported that tentacle expansion and contraction behavior differed among coral types. The tentacles of *Favia favus* completely contracted under exposure to light at 400–520 nm and 540–700 nm (10 μmol quanta m^−2^s^−1^), contracted under exposure to light at 660–700 nm (30 μmol quanta m^−2^s^−1^) light, and extended in the absence of light. However, *Globorotalia lobata*, *S. pistillata*, and *Centruroides gracilis* did not respond to light at any of the wavelengths tested. *Plerogyra sinuosa* was exposed to light at 400–540 nm (30 μmol quanta m^−2^s^−1^). After 1–2 days, the tentacles contracted completely [10]. We observed that *G. columna* achieved polyp extension rapidly under either LP or LB light at 400–470 nm (68–70 μmol m^−2^s^−1^), with partial extension within 5 min and 100% extension in 30 min. By contrast, the tentacles of *G. columna* illuminated by LG or LB at 500–650 nm (45–50 μmol m^−2^s^−1^) only began to extend after 10 min, reaching 25% extension after 30 min. Furthermore, polyp shrinkage was observed in the LR and LG groups after the experiment. Therefore, our study demonstrated that red light (620–650 nm) and green light (500–540 nm) were unsuitable for *G. columna* cultivation. The most suitable wavelengths for *G. columna* growth were determined to be LB (440–470 nm) and LP (400–430 nm).

Heterotrophic feeding is essential for coral nutrition because corals and symbiotic zooxanthellae obtain basic nutrients through plankton capture [10]. A previous study revealed that heterotrophy can stimulate coral growth and increase tissue protein concentration in *S. pistillata* [10,47]. The heterotrophic feeding of corals is affected by light. Ferrier-Pages et al. [48] indicated that as light intensity gradually increases [49], the ingestion rate of *S. pistillata* declines and the polyps fully swell in the dark; by contrast, they usually remain contracted under light exposure [50]. The feeding ability of *P. damicornis* is not affected by short-term exposure to dark conditions, and *Galaxea fascicularis* exhibits no significant differences in ingestion rate in light and darkness. However, *G. fascicularis* fed *Artemia nauplii* quickly reach saturation under illumination [51]. In addition, studies have suggested that the coral mesentery is a key part of the digestive process, secreting digestive enzymes for extracellular digestion and enabling the ingestion, digestion, and absorption of food [52,53]. In the wild, the protein content of *Montastraea faveolata*, a stony coral, accounts for 5–9% of the total weight of the coral, on average; however, the protein content of the soft coral *Pachyclavularia violacea* accounts for 8–12% of the weight of the coral [54]. We determined that protein accounted for 6–8% of the non-skeletal tissue weight of *G. columna*. Digestive enzyme activity is necessary for nutritional digestion and absorption and may regulate growth in various species [55,56], but previous research has not reported such activity in corals. Ding et al. [2] found that *G. columna* digestive enzymes and body composition would change due to photoperiod and feed. According to body composition and digestive enzyme experiments, it was found that the protein content of corals was highest at 12:00, and significantly decreased at 18:00, 00:00 and 6:00 (the next day). Digestive enzyme protease activity reached the highest at 12:00, decreased 1.35 times at 18:00, and reached the lowest at 6:00 the next day. Therefore, feeding at suitable light wavelengths can enhance protease activity and facilitate feeding and nutrient absorption. In addition, different light sources also have physiological adjustment functions for corals. Levy et al. [57] found that *Acropora millepora* have cryptochrome genes capable of sensing blue light, so corals can regulate physiological time and adjust spawning time by sensing moonlight regulation.

In this study, *G. columna* had the lowest protease activity and protein content in darkness and the highest protease activity and protein content under LP and LB illumination. Lipase activity and amylase activity did not differ significantly among the groups, and zooxanthellae and chlorophyll a concentrations did not significantly differ among the illumination groups but decreased significantly in the control group. Therefore, our findings suggest that light may enhance protease activity and protein content of *G. columna* symbionts under at least 6 h of daily LB (440–470 nm) and LP (400–430 nm) exposure.

## 5. Conclusions

Our findings indicate that light is crucial for *G. columna* growth although no significant effects on zooxanthellae density or chlorophyll a concentration were observed. At least 6 h per day LB or LP (400–470 nm) light and feeding will help growth, polyp extension, protein composition, and protease activity. For aquaculture applications, shortening the light time will reduce the cost of electricity. These are the optimal conditions for aquaculture *G. columna*. In addition to being applicable to large-scale farming, our findings can reduce the collection of wild corals and allow natural coral to achieve conservation and repopulation. At present, the light exposure at the CITES-certified Taiwan Coral King coral farm (Kaohsiung, Taiwan) is at wavelengths of 400–470 nm. Moreover, to conserve energy, daily light exposure can be reduced to 6 h. Under these conditions, the annual output of *G. columna* colonies in 150 × 60 × 30 cm glass tank can reach 3000.

## 6. Patents

This research achievement has applied for two Taiwan patents: the invention patent number is I602505 and the utility model patent number is M551414.

## Figures and Tables

**Table 1:** Wavelengths of light sources.

Exposure Times	Light	Wavelength of Light (nm)	Photosynthetically Active Radiation (μmol m^−2^s^−1^)
0 h	C	Dark	0
6 h	LW	444–696	102.23 ± 1.01
	LY	570–590	53.63 ± 0.32
	LR	620–650	44.05 ± 1.01
	LG	500–540	46.35 ± 0.75
	LB	440–470	66.67 ± 0.38
	LP	400–430	71.03 ± 0.21
12 h	LW	444–696	100.58 ± 0.73
	LY	570–590	54.02 ± 0.57
	LR	620–650	43.98 ± 1.31
	LG	500–540	48.49 ± 1.54
	LB	440–470	68.03 ± 0.42
	LP	400–430	70.49 ± 0.44

Values represent means ± SD (*n* = 56 day). C, Dark; LW, White light; LG, Green light; LY, Yellow light; LB, Blue light; LR, Red light; LP, Purple light. Different letters indicate significant differences among groups (*p* < 0.05). Values are expressed as means ± SDs (*n* = 30).

**Table 2:** Water quality conditions.

		6 h						12 h					
WaterQuality Conditions	C	LW	LY	LG	LR	LB	LP	LW	LY	LG	LR	LB	LP
Temperature (°C)	26.23 ± 0.73	26.15 ± 0.23	26.23 ± 0.71	26.41 ± 0.52	26.22 ± 0.25	26.41 ± 0.32	26.04 ± 0.43	26.91 ± 0.33	26.48 ± 0.64	26.32 ± 0.31	26.93 ± 0.34	26.63 ± 0.30	26.42 ± 0.21
Salinity (PSU)	35.12 ± 0.42	35.50 ± 0.31	35.24 ± 0.12	35.42 ± 0.38	34.82 ± 0.92	35.41 ± 0.91	35.73 ± 0.52	35.25 ± 0.49	35.33 ± 0.23	34.41 ± 0.52	35.23 ± 0.21	35.02 ± 0.38	34.94 ± 0.32
pH	8.01 ± 0.41	8.05 ± 0.29	8.28 ± 0.32	8.03 ± 0.31	8.32 ± 0.91	8.21 ± 0.42	8.01 ± 0.21	8.26 ± 0.31	8.03 ± 0.33	8.02 ± 0.48	8.14 ± 0.93	8.03 ± 0.31	8.32 ± 0.53
Ammonia nitrogen (mg/L)	0.04 ± 0.02	0.04 ± 0.02	0.04 ± 0.01	0.05 ± 0.02	0.04 ± 0.01	0.04 ± 0.02	0.03 ± 0.05	0.04 ± 0.02	0.03 ± 0.01	0.03 ± 0.01	0.02 ± 0.01	0.03 ± 0.01	0.03 ± 0.02
Nitrous acid (mg/L)	0.02 ± 0.01	0.02 ± 0.01	0.02 ± 0.01	0.01 ± 0.01	0.01 ± 0.01	0.02 ± 0.01	0.02 ± 0.01	0.02 ± 0.01	0.02 ± 0.01	0.02 ± 0.02	0.02 ± 0.01	0.02 ± 0.01	0.01 ± 0.01
Nitric acid (PPM)	0.15 ± 0.04	0.15 ± 0.04	0.21 ± 0.02	0.19 ± 0.02	0.09 ± 0.03	0.20 ± 0.03	0.21 ± 0.02	0.22 ± 0.05	0.21 ± 0.02	0.18 ± 0.02	0.20 ± 0.04	0.19 ± 0.03	0.19 ± 0.02
Calcium (PPM)	400 ± 43.21	410 ± 43.21	410 ± 15.27	408 ± 18.21	403 ± 21.32	415 ± 17.62	412 ± 9.01	432 ± 32.53	421 ± 14.42	410 ± 31.21	408 ± 31.31	414 ± 3.21	422 ± 17.21
Magnesium (PPM)	1280 ± 54.42	1280 ± 54.42	1289 ± 32.12	1300 ± 21.32	1281 ± 15.21	1321 ± 21.21	1332 ± 21.37	1321 ± 22.01	1298 ± 31.26	1320 ± 11.23	1320 ± 10.32	1318 ± 11.35	1299 ± 9.32
Phosphate (PPM)	0.02 ± 0.01	0.02 ± 0.01	0.01 ± 0.01	0.02 ± 0.01	0.02 ± 0.01	0.02 ± 0.01	0.02 ± 0.01	0.02 ± 0.01	0.01 ± 0.01	0.02 ± 0.01	0.02 ± 0.01	0.02 ± 0.01	0.02 ± 0.01

Values represent means ± SD (*n* = 56 day). C, Dark; LW, White light; LG, Green light; LY, Yellow light; LB, Blue light; LR, Red light; LP, Purple light. Different letters indicate significant differences among groups (*p* < 0.05). Values are expressed as means ± SDs (*n* = 30). 6 h: 6 h exposure, 12 h: 12 h exposure.

**Table 3:** Scores for stretching and contractile behaviors in *Goniopora columna* polyps.

Stretching and Contractile	Stretching and Contractile Behaviors	Score
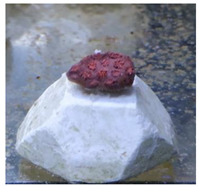	Complete contraction	0
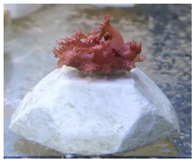	When the polyps were elongated slightly	1
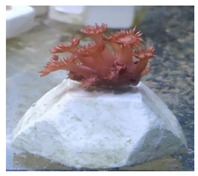	When some polyps, but less than half in total (50%), were extended	2
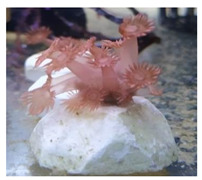	When most polyps, more than half in total (75%), were extended	3
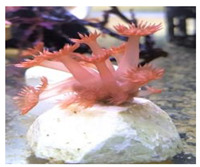	When all polyps were fully extended (100%)	4

**Table 4:** Body composition of *Goniopora columna* after 8 weeks of daily exposure to different light sources for 6 or 12 h.

Nutritional Indicators	Treatments												
	C	LW		LY		LR		LG		LB		LP	
		6 h	12 h	6 h	12 h	6 h	12 h	6 h	12 h	6 h	12 h	6 h	12 h
Protein (µg)	226.44 ± 7.08 ^f^	366.95 ± 7.00 ^c^	372.13 ± 1.21 ^c^	357.21 ± 6.70 ^d^	343.25 ± 3.40 ^d^	224.12 ± 4.42 ^f^	234.10 ± 3.74 ^f^	263.99 ± 7.32 ^e^	276.32 ± 6.24 ^e^	390.06 ± 3.38 ^b^	395.06 ± 3.09 ^b^	426.61 ± 6.42 ^a^	421.11 ± 2.32 ^a^
Lipid (µg)	1.59 ± 0.21	1.39 ± 0.47	1.35 ± 0.16	1.51 ± 0.12	1.32 ± 0.35	0.98 ± 0.34	1.03 ± 0.32	1.29 ± 0.18	1.45 ± 0.12	1.64 ± 0.27	1.35 ± 0.15	1.66 ± 0.66	1.43 ± 0.25
Glucose (µg)	1.30 ± 0.21	1.37 ± 0.58	1.28 ± 0.24	1.32 ± 0.41	1.28 ± 0.34	1.33 ± 0.37	1.22 ± 0.26	1.39 ± 0.22	1.28 ± 0.13	1.34 ± 0.17	1.48 ± 0.14	1.58 ± 0.19	1.59 ± 0.12

SD, standard deviation. C, Dark; LW, White light; LG, Green light; LY, Yellow light; LB, Blue light; LR, Red light; LP, Purple light. Different letters indicate significant differences among groups (*p* < 0.05). Values are expressed as means ± SDs (*n* = 30).

**Table 5:** Activity of digestive enzymes in *Goniopora columna* after 8 weeks of daily exposure to different light sources for 6 or 12 h.

Treatments													
Test Items	C	LW		LY		LR		LG		LB		LP	
		6 h	12 h	6 h	12 h	6 h	12 h	6 h	12 h	6 h	12 h	6 h	12 h
Protease (U/mg protein)	58.55 ± 5.83 ^g^	169.04 ± 4.11 ^c^	197.32 ± 3.42 ^b^	155.20 ± 8.45 ^d^	159.12 ± 5.24 ^d^	97.27 ± 2.49 ^f^	98.21 ± 1.89 ^f^	114.01 ± 2.56 ^e^	109.01 ± 3.54 ^e^	191.65 ± 3.91 ^b^	184.02 ± 5.38 ^b^	231.37 ± 9.00 ^a^	237.21 ± 5.40 ^a^
Lipase (U/mg protein)	7.53 ± 0.85 ^d^	9.77 ± 0.58 ^c^	9.85 ± 0.67 ^c^	9.05 ± 0.37 ^c^	8.37 ± 0.45 ^d^	6.73 ± 0.56 ^e^	7.21 ± 0.49 ^e^	6.04 ± 1.36 ^e^	5.03 ± 1.42 ^e^	11.90 ± 1.00 ^b^	9.53 ± 0.87 ^c^	12.05 ± 1.74 ^ab^	14.24 ± 1.31 ^a^
Amylase (U/mg protein)	1.77 ± 0.12	1.95 ± 0.32	1.89 ± 0.17	1.87 ± 0.12	1.96 ± 0.43	1.73 ± 0.14	1.84 ± 0.43	1.54 ± 0.16	1.65 ± 0.12	1.93 ± 0.38	1.57 ± 0.24	1.90 ± 0.26	2.03 ± 0.19

SD, standard deviation. C, Dark; LW, White light; LG, Green light; LY, Yellow light; LB, Blue light; LR, Red light; LP, Purple light. Different letters indicate significant differences among groups (*p* < 0.05). Values are expressed as means ± SDs (*n* = 30).

**Table 6:** Zooxanthellae density and chlorophyll a concentration in *G. columna* after 8 weeks of daily exposure to different light sources for 6 or 12 h.

Exposure Times	Treatments	Zooxanthellae (Cells × 10^7^ m^−2^)	Chlorophyll a (µg cm^−^^2^)
0 h	C	0.3 ± 0.13 ^b^	14 ± 1.33 ^b^
6 h	LW	3.9 ± 1.74 ^a^	52 ± 3.72 ^a^
	LY	3.9 ± 0.73 ^a^	51 ± 2.53 ^a^
	LR	3.9 ± 1.18 ^a^	51 ± 4.52 ^a^
	LG	3.9 ± 1.02 ^a^	50 ± 2.34 ^a^
	LB	4.0 ± 1.32 ^a^	52 ± 4.21 ^a^
	LP	4.0 ± 0.81 ^a^	52 ± 3.42 ^a^
12 h	LW	4.0 ± 2.14 ^a^	53 ± 5.21 ^a^
	LY	3.9 ± 2.13 ^a^	51 ± 3.04 ^a^
	LR	3.9 ± 2.51 ^a^	51 ± 4.01 ^a^
	LG	3.9 ± 3.23 ^a^	50 ± 5.02 ^a^
	LB	4.0 ± 2.64 ^a^	53 ± 3.84 ^a^
	LP	4.0 ± 1.92 ^a^	53 ± 3.02 ^a^

SD, standard deviation. C, Dark; LW, White light; LG, Green light; LY, Yellow light; LB, Blue light; LR, Red light; LP, Purple light. Different letters indicate significant differences among groups (*p* < 0.05). Values are expressed as means ± SDs (*n* = 30).

## Data Availability

Not applicable.
